# Taxonomic notes on the crane fly genus *Thrypticomyia* Skuse (Diptera, Limoniidae) from Chinese Mainland, with description of a new species

**DOI:** 10.3897/BDJ.13.e159998

**Published:** 2025-09-11

**Authors:** Liying Dai, Shuo Ma, Yufei Zhao, Xiao Zhang

**Affiliations:** 1 Shandong Engineering Research Center for Environment-Friendly Agricultural Pest Management, College of Plant Health and Medicine, Qingdao Agricultural University, Qingdao 266109, China Shandong Engineering Research Center for Environment-Friendly Agricultural Pest Management, College of Plant Health and Medicine, Qingdao Agricultural University Qingdao 266109 China

**Keywords:** Chinese fauna, distribution, new species, taxonomy

## Abstract

**Background:**

*Thrypticomyia* Skuse, 1890 is a limoniid genus with 38 known species and subspecies from the Australasian, Oriental, Afrotropic and Palaearctic Regions, of which two are recorded in Chinese Mainland.

**New information:**

A *Thrypticomyia* species from Sichuan, China, *T. sichuanensis* sp. nov., is described and illustrated as new to science. The genus *Thrypticomyia* is recorded from Sichuan for the first time.

## Introduction

*Thrypticomyia* Skuse, 1890 is a small genus in the family Limoniidae (Diptera) with 38 species and subspecies worldwide, of which 24 are from the Australasian Region, 12 from the Oriental Region, five are from the Afrotropical Region and one is from the Palaearctic Region (Oosterbroek 2025). The genus can be well defined by the white tarsi of legs, the wing without microtrichia, the vein CuA lacking and the vein R_3_ with distinct spur (Alexander 1972, Skuse 1890). Some other limoniid crane flies also have similar features on their legs, such as the subgenera Elephantomyia (Elephantomyodes) Alexander, 1923 and Dicranomyia (Euglochina) Alexander, 1921, but *Thrypticomyia* can be easily distinguished from them by the features on the wing mentioned before (Alexander 1921a, Alexander 1923). Members of the *Thrypticomyia* can dance with placing the ends of their fore tarsi on a spider-web line and sometimes they rest close together on a line (Alexander 1927a, Alexander 1931, Alexander 1972, Savchenko and Krivolutskaya 1976, Podenas et al. 2017). Some species were found ovipositing in green slime in a small ditch (Edwards 1928).

Currently, only two *Thrypticomyia* crane flies have been known to occur in Chinese Mainland, namely, *T. apicalis apicalis* (Wiedemann, 1828) and *T. unisetosa unisetosa* (Alexander, 1929), which were first published as new species from Indonesia and Japan, respectively. In this study, taxonomic notes for the two known subspecies, based on specimens and literature, are provided. In addition, a new *Thrypticomyia* species from Sichuan, China, *T. sichuanensis* sp. nov., is described and illustrated, representing the first record of this genus in Sichuan.

## Materials and methods

Studied specimens of *T. sichuanensis* sp. nov. and *T. unisetosa unisetosa* (Alexander, 1929) are deposited in QAU (the Entomological Museum of Qingdao Agricultural University, Shandong, China). Studied specimens of *T. apicalis apicalis* (Wiedemann, 1828), which were identified by C.P. Alexander, are deposited in USNM (the National Museum of Natural History, Smithsonian Institution, Washington D.C., USA). The specific preparation work for male is macerating the apical portion of the abdomen in cold 10% sodium hydroxide (NaOH) for 12–15 hours. Photographs were taken by using a Canon EOS 90D digital camera through a macro lens.

The morphological terminology mainly follows Cumming and Wood (2017) and de Jong (2017). The following abbreviations in figures are used: aed = aedeagus, goncx = gonocoxite, i gonst = inner gonostylus, o gonst = outer gonostylus, pm = paramere, rp = rostral prolongation, tg 9 = tergite 9.

## Taxon treatments

### Thrypticomyia apicalis apicalis

(Wiedemann, 1828)

0F635603-C276-58FD-BBD2-967684AD158C

Limnobia apicalis Wiedemann, 1828. Wiedemann (1828): 551. Type locality: Indonesia: Sumatra.
Dicranomyia (Thrypticomyia) apicalis Wiedemann, 1828. Alexander (1927a): 298.

#### Materials

- **Type status:** Other material. **Occurrence:** recordedBy: Gressitt; individualCount: 1; sex: male; occurrenceID: E3859565-7559-5635-B65E-609A68B23034; **Taxon:** class: Insecta; order: Diptera; family: Limoniidae; genus: Thrypticomyia; **Location:** country: China; stateProvince: Hainan; locality: Ta Han; **Event:** year: 1935; month: 7; day: 22; **Record Level:** institutionCode: USNM
- **Type status:** Other material. **Occurrence:** recordedBy: Lund; individualCount: 1; sex: male; occurrenceID: 42ECECB2-FD01-5F3A-835B-5BA9E5C23985; **Taxon:** class: Insecta; order: Diptera; family: Limoniidae; genus: Thrypticomyia; **Location:** country: Sri Lanka; stateProvince: Western; **Event:** year: 1942; month: 1; day: 21; **Record Level:** institutionCode: USNM

#### Diagnosis

Antenna dark brown. Prescutum and presutural scutum dark brown with lateral margins paler. Pleuron pale yellow, darker near base of wing. Basitarsi of legs white with basal 1/4–2/5 dark brown, remainder of tarsi white, except terminal, pale brown ends. Wing with tip distinctly darkened. Sc ending just beyond origin of Rs; sc-r far away from base of Rs; R_2_ long, about 2.5 times length of R_2+3_; R_3_ spur slightly longer than R_2+3_; m-cu near 2/3 of dm; distal end of CuA slightly longer than m-cu. Gonocoxite with a ventromesal lobe at middle, the lobe about 2/3 length of gonocoxite. Inner gonostylus slightly longer than gonocoxite; rostral prolongation about 2/3 width of inner gonostylus; two spines at base of rostral prolongation straight and long, widely separated from each other, more basal one arising from a tubercle. Paramere horn-shaped and curved outwards.

#### Distribution

China (Hainan); Indonesia, Malaysia, Philippines, Sri Lanka.

#### Notes

When this species was first published by Wiedemann (1828), only a very brief description was provided. Alexander (1927a) re-described holotype of this species and provided a more detailed description with illustrations of wing and male hypopygium based on other specimens. In the same year, he added a complete illustration of the wing (Alexander 1927b). Alexander (1930) published the new subspecies majuscula for this species, which can be easily distinguished by the larger body size (Alexander 1930).

### Thrypticomyia unisetosa unisetosa

(Alexander, 1929)

FAA10B8B-EB5A-5817-AAF3-ED6EC0AECA40

Thrypticomyia arcuata Alexander, 1920. Alexander (1920): 4. Type locality: Japan: Tokyo.
Limonia (Thrypticomyia) unisetosa Alexander, 1929. Alexander (1929a): 248.
Thrypticomyia unisetosa Alexander, 1929. Savchenko and Krivolutskaya (1976): 123.
Thrypticomyia unisetosa unisetosa Alexander, 1929. Podenas et al. (2017): 275.

#### Materials

- **Type status:** Other material. **Occurrence:** recordedBy: Xingyang Qian; individualCount: 3; sex: 2 males, 1 female; lifeStage: adult; occurrenceID: C7A6676A-7D4E-50DF-93C8-CCB7F6A3EB00; **Taxon:** class: Insecta; order: Diptera; family: Limoniidae; genus: Thrypticomyia; **Location:** country: China; stateProvince: Zhejiang; county: Suichang; locality: Jiulongshan National Nature Reserve, Jiulongkou Village; **Event:** samplingProtocol: insect net; year: 2019; month: 7; day: 24; **Record Level:** institutionCode: QAU
- **Type status:** Other material. **Occurrence:** recordedBy: Xingyang Qian; individualCount: 2; sex: 1 male, 1 female; lifeStage: adult; occurrenceID: C645A1A4-4FE2-5957-8252-C765DC2C400D; **Taxon:** class: Insecta; order: Diptera; family: Limoniidae; genus: Thrypticomyia; **Location:** country: China; stateProvince: Zhejiang; county: Suichang; locality: Jiulongshan National Nature Reserve, Jiulongkou Village; **Event:** samplingProtocol: light trap; year: 2019; month: 7; day: 24; **Record Level:** institutionCode: QAU

#### Diagnosis

Antenna dark brown with scape and pedicel brown. Prescutum and presutural scutum brown with anterior margin dark brown and lateral margins paler. Pleuron pale yellow, darker near base of wing. Basitarsi of legs white with basal 1/10–1/8 black, remainder of tarsi white, except terminal, pale brown ends. Wing with tip very indistinctly darkened. Sc ending just beyond origin of Rs; sc-r far away from base of Rs; R_2_ ranged from as long as R_2+3_ to 2.5 times length of R_2+3_; R_3_ spur ranged from slightly shorter than R_2+3_ to twice length of R_2+3_; m-cu slightly beyond middle of dm; distal end of CuA nearly as long as m-cu. Gonocoxite with a ventromesal lobe at apical 1/3, the lobe slightly longer than length of gonocoxite. Inner gonostylus about 2/3–3/4 length of gonocoxite; rostral prolongation about half width of inner gonostylus, spines very small to virtually lacking. Paramere hook-shaped and curved inwards, subtip slightly inflated.

#### Distribution

China (southeast, Zhejiang, Taiwan); Russia, North Korea, South Korea, Japan.

#### Notes

This species was first published as a new species by Alexander (1920) under the name *arcuata*, which was later replaced by the new name *unisetosa* (Alexander 1929a). Savchenko and Krivolutskaya (1976) added an illustration of male hypopygium for this species. Podenas et al. (2017) provided detailed description with illustrations of antenna, wing, male hypopygium and female ovipositor. The two subspecies nigribasis and *perelongata* of this species were published by Alexander (1958) and Alexander (1972), respectively, with the main difference being the significantly increased black areas on the basitarsi of legs. The nominotypical subspecies can be distinguished from other subspecies by the basal 1/10–1/8 of the basitarsi being black. In *nigribasis* and *perelongata*, the basal 1/4 or more of the basitarsi are black (Alexander 1958, Alexander 1972). Oosterbroek (2025) indicated that this species was distributed in south-eastern China, but the specific province was not given. This study provides a specific province (i.e. Zhejiang) where this species is distributed in south-eastern China.

### Thrypticomyia sichuanensis

Dai & Zhang sp. nov.

784BF65F-3485-5897-BC78-AB95BF4F0901

4C6FFEEA-A1D5-44B4-8210-6B6CE48EA7A5

#### Materials

- **Type status:** Holotype. **Occurrence:** recordedBy: Liang Wang; individualCount: 1; sex: male; occurrenceID: 136E473D-3C3F-5036-A2D8-5647A0158C51; **Taxon:** class: Insecta; order: Diptera; family: Limoniidae; genus: Thrypticomyia ; **Location:** country: China; stateProvince: Sichuan; county: Yanyuan; locality: Lugu Lake, Dujia Village; verbatimElevation: 2700 m; **Event:** samplingProtocol: insect net; year: 2019; month: 7; day: 20; **Record Level:** institutionCode: QAU
- **Type status:** Paratype. **Occurrence:** recordedBy: Liang Wang; individualCount: 4; sex: male; occurrenceID: F0207CFA-EEFB-5498-B372-6CB0CED33D6C; **Taxon:** class: Insecta; order: Diptera; family: Limoniidae; genus: Thrypticomyia ; **Location:** country: China; stateProvince: Sichuan; county: Yanyuan; locality: Lugu Lake, Dujia Village; verbatimElevation: 2700 m; **Event:** samplingProtocol: insect net; year: 2019; month: 7; day: 20; **Record Level:** institutionCode: QAU
- **Type status:** Paratype. **Occurrence:** recordedBy: Liang Wang; individualCount: 2; sex: male; occurrenceID: AB0179DB-09BB-5410-89E1-535FE83D5699; **Taxon:** class: Insecta; order: Diptera; family: Limoniidae; genus: Thrypticomyia ; **Location:** country: China; stateProvince: Sichuan; county: Muli; locality: Liziping; verbatimElevation: 2370 m; **Event:** samplingProtocol: insect net; year: 2019; month: 7; day: 26; **Record Level:** institutionCode: QAU

#### Description

**Diagnosis.** Antenna dark brown. Prescutum and presutural scutum brown with anterior margin dark brown, posterior half with two narrow pale lines. Pleuron pale yellow, darker near base of wing. Basitarsi of legs white with basal 1/12–1/10 black, remainder of tarsi white, except terminal, pale brown ends. Wing with Sc ending beyond origin of Rs and opposite about 1/6 of Rs; sc-r at base of Rs; R_2_ very short, about 1/4 length of R_2+3_; R_3_ spur slightly longer than R_2+3_; m-cu slightly beyond middle of dm; distal end of CuA nearly as long as m-cu. Gonocoxite with a ventromesal lobe at the middle, the lobe slightly longer than half length of gonocoxite. Inner gonostylus about or slightly longer than gonocoxite; rostral prolongation about half width of inner gonostylus, basally bearing two spines. Paramere hook-shaped and curved inwards.

**Male**. Body length 5.0–6.0 mm, wing length 6.5–8.0 mm, halter length 1.2–1.5 mm.

**Figure 1.**
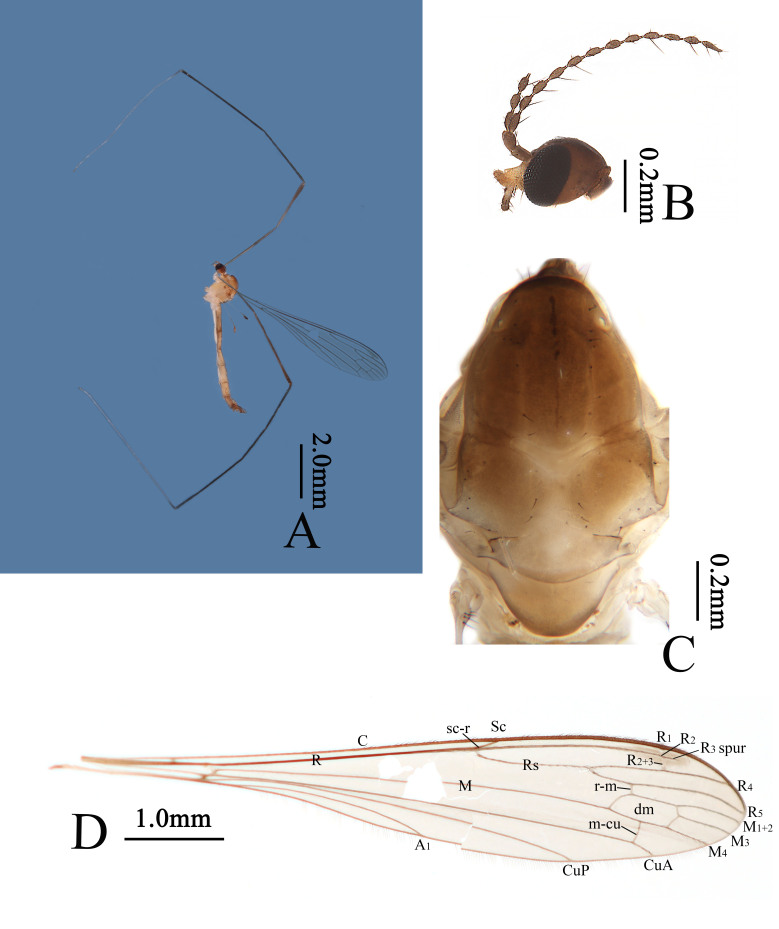
*Thrypticomyia sichuanensis* sp. nov. **A** habitus of male, lateral view; **B** head, lateral view; **C** thorax, dorsal view; **D** wing.

Head (Fig. 1A, B). Dark brown. Setae on head brownish-black. Antenna dark brown throughout. Scape cylindrical, two times as long as wide. Pedicel oval. Flagellomeres long-oval, each with an apical stalk and a single long dark brown bristle. Rostrum pale yellow with dark brown setae. Palpus greyish-brown with dark brown setae.

Thorax (Fig. 1A, C). Pronotum dark brown. Prescutum and presutural scutum brown with anterior margin dark brown, posterior half with two narrow pale lines. Postsutural scutum brown with middle area pale, each lobe with a pale spot. Scutellum greyish-yellow with posterior margin pale brown. Mediotergite pale brown with posterior margin brown, both sides of base pale. Pleuron (Fig. 1A) pale yellow, darker near base of wing. Setae on thorax dark brown. Coxae greyish-yellow; trochanters white; femora brown to dark brown; tibiae black; basitarsi white with basal 1/12–1/10 black, remainder of tarsi white, except terminal ends pale brown. Setae on legs dark brown. Wing (Fig. 1D) pale brown; stigma long-oval, brown. Vein brown. Venation: Sc ending beyond origin of Rs and opposite about 1/6 of Rs; sc-r at base of Rs; Rs long, about 4/5 of total length of R_2+3+4_ and R_4_; R_2_ very short, about 1/4 length of R_2+3_; R_3_ spur slightly longer than R_2+3_; m-cu slightly beyond middle of dm; distal end of CuA nearly as long as m-cu. Halter brownish-black with basal 3/5 of stem pale yellow.

Abdomen (Fig. 1A). Tergites 1–8 brownish-yellow to pale brown, sternites 1–8 white. Segment 9 brown. Setae on abdomen dark brown.

**Figure 2.**
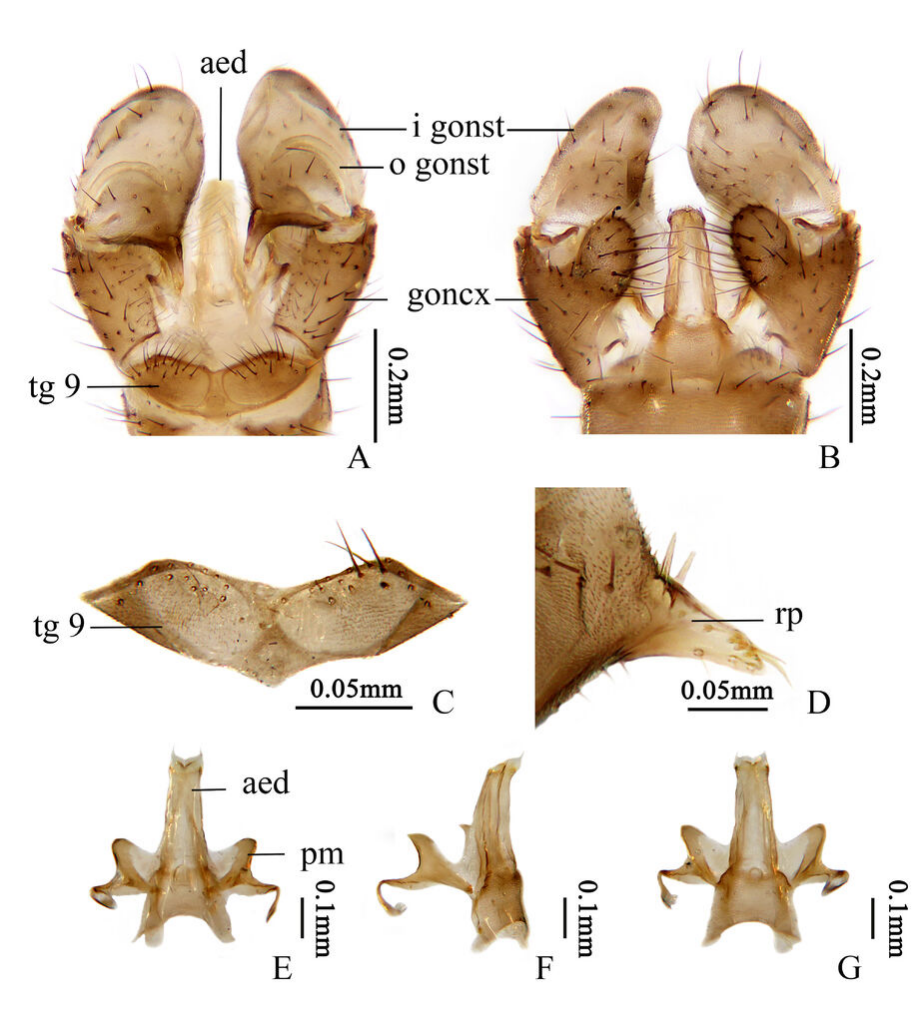
*Thrypticomyia sichuanensis* sp. nov. **A** male hypopygium, dorsal view; **B** male hypopygium, ventral view; **C** tergite 9, dorsal view; **D** rostral prolongation of inner gonostylus, dorsal view; **E** aedeagal complex, dorsal view; **F** aedeagal complex, lateral view; **G** aedeagal complex, ventral view.

Hypopygium (Fig. 2). Pale brown to brown. Tergite 9 (Fig. 2A, C) transverse, posterior margin with a shallow emargination, divided into two lobes, each with about 15–18 long, dark brown setae. Gonocoxite (Fig. 2A, B) cylindrical with short, dark brown setae, middle with a ventromesal lobe; the lobe of moderate length, slightly longer than half length of gonocoxite, inner edge with dense long dark brown setae. Outer gonostylus (Fig. 2A) curved, with tip acute. Inner gonostylus (Fig. 2A, B) large and fleshy, about or slightly longer than gonocoxite; rostral prolongation long and curved, about half width of inner gonostylus; base broad, provided with two straight spines (Fig. 2D). Paramere (Fig. 2E-G) slightly sclerotised, hook-shaped and curved inwards; a membranous structure connecting inner sides of two parameres and base of aedeagus (Fig. 2E, G). Aedeagus (Fig. 2E-G) long, widened at base, each side of apex with a short transparent spine.

#### Etymology

The specific epithet "*sichuanensis*" (adjective) refers to the type locality Sichuan.

#### Distribution

China (Sichuan).

#### Notes

From two others *Thrypticomyia* species known in Chinese Mainland, *T. sichuanensis* sp. nov. can be easily distinguished by the wing having no darkened tip, while in *T. apicalis apicalis* (Wiedemann, 1828) and *T. unisetosa unisetosa* (Alexander, 1929), the tip of the wing is more or less darkened. The new species can be further distinguished by the inner gonostylus bearing two spines on the rostral prolongation. The two known species, *T. apicalis apicalis* (Wiedemann, 1828) and *T. unisetosa unisetosa* (Alexander, 1929), can be easily distinguished by the length of the ventromesal lobe on the gonocoxite of male hypopygium.

The new species *T. sichuanensis* sp. nov. is similar to *T. octosetosa* (Alexander, 1931) from Philippines in having similar black areas on the basitarsi of legs, but it can be distinguished by the wing with Sc ending beyond the origin of Rs and opposite about 1/6 of Rs (Fig. 1D) and the tergite 9 with a shallow emargination at posterior margin and 15–18 long setae on each lobe (Fig. 2A, C). In *T. octosetosa* (Alexander, 1931), the vein Sc ends before the origin of Rs and the tergite 9 has a deep notch at the posterior margin and four powerful setae on each lobe (Alexander 1931).

## Identification Keys

### Key to species and subspecies of *Thrypticomyia* from the Palaearctic and Oriental Regions

**Table:** 

1	Wing with tip distinctly darkened.	2
–	Wing with tip not darkened or very indistinctly darkened.	3
2	Body size small, about 5.0 mm; basitarsi of fore legs white with basal 2/5 darkened; rostral prolongation of inner gonostylus with two spines widely separated (Wiedemann 1828, Alexander 1927a).	*Thrypticomyia apicalis apicalis* (Wiedemann, 1828)
–	Body size large, about 7.0–7.5 mm; basitarsi of fore legs white with basal 1/5 blackened; rostral prolongation of inner gonostylus with two spines slightly separated (Alexander 1930).	*Thrypticomyia apicalis majuscula* (Alexander, 1930)
3	Wing with cell dm open (Alexander 1967a).	*Thrypticomyia aclistia* (Alexander, 1967)
–	Wing with cell dm closed.	4
4	Wing without stigma (Alexander 1967b).	*Thrypticomyia estigmata* (Alexander, 1967)
–	Wing with stigma.	5
5	Ventromesal lobe of gonocoxite exceeding top of inner gonostylus.	6
–	Ventromesal lobe of gonocoxite not exceeding top of inner gonostylus.	8
6	Basitarsi of legs white with basal 1/2 or more black; wing with Sc₁ ending at origin of Rs (Alexander 1972).	*Thrypticomyia unisetosa perelongata* (Alexander, 1972)
–	Basitarsi of legs white with basal 1/3 or less black; wing with Sc₁ ending beyond origin of Rs.	7
7	Basitarsi of legs white with basal 1/4–1/3 black (Alexander 1958).	*Thrypticomyia unisetosa nigribasis* (Alexander, 1958)
–	Basitarsi of legs white with basal 1/10–1/8 black (Alexander 1929a).	*Thrypticomyia unisetosa unisetosa* (Alexander, 1929)
8	Wing with Sc₁ ending at or beyond origin of Rs.	9
–	Wing with Sc₁ ending before origin of Rs.	10
9	Basitarsi of legs white with basal 2/5 dark brown; wing with Sc₁ ending at origin of Rs and m-cu before mid-length of cell dm (Alexander 1929b).	*Thrypticomyia brevicuspis* (Alexander, 1929)
–	Basitarsi of legs white with basal 1/12–1/10 black; wing with Sc₁ ending beyond origin of Rs and m-cu beyond mid-length of cell dm.	*Thrypticomyia sichuanensis* sp. nov.
10	Sc₁ very short, ending far before the origin of Rs (Alexander 1921b).	*Thrypticomyia microstigma* (Alexander, 1921)
–	Sc₁ normal, ending slightly before the origin of Rs.	11
11	Rostral prolongation of inner gonostylus with a single spine (Alexander 1927b).	*Thrypticomyia monocera* (Alexander, 1927)
–	Rostral prolongation of inner gonostylus with two spines.	12
12	Wing with Rs angulated, sometimes short-spurred; spines on rostral prolongation of inner gonostylus relatively short with basal one arising from an enlarged tubercle (Alexander 1927a).	*Thrypticomyia arachnophila* (Alexander, 1927)
–	Wing with Rs curved; spines on rostral prolongation of inner gonostylus small with basal enlargement very small to virtually lacking (Alexander 1931).	*Thrypticomyia octosetosa* (Alexander, 1931)

## Supplementary Material

XML Treatment for Thrypticomyia apicalis apicalis

XML Treatment for Thrypticomyia unisetosa unisetosa

XML Treatment for Thrypticomyia sichuanensis
